# Beyond Diffusion Capacity: Continuous Exercise Oximetry Reveals Phenotype-Related Cardiopulmonary Signals in Idiopathic Pulmonary Fibrosis and Progressive Pulmonary Fibrosis—A eurILDreg Pilot Study

**DOI:** 10.3390/jcm15145572

**Published:** 2026-07-16

**Authors:** Silke Tello, Anita C. Windhorst, Nadia Hamadi, Andreas Guenther, Ekaterina Krauss

**Affiliations:** 1 European IPF/ILD Registry & Biobank (eurIPFreg/Bank, eurILDreg/Bank), 35392 Giessen, Germany; 2 Center for Interstitial and Rare Lung Diseases, Universities of Giessen and Marburg Lung Center (UGMLC), Justus-Liebig University Giessen, Member of the German Center for Lung Research (DZL), 35392 Giessen, Germany; 3 Institute of Medical Informatics, Justus-Liebig University, 35392 Giessen, Germany; 4 Agaplesion Lung Clinic, Evangelisches Krankenhaus Mittelhessen, Paul-Zipp Str. 171, 35398 Giessen, Germany; 5 Institute for Lung Health (ILH), 35392 Giessen, Germany; 6 Cardio-Pulmonary Institute (CPI), Klinikstr. 33, 35392 Giessen, Germany

**Keywords:** interstitial lung disease, idiopathic pulmonary fibrosis, progressive pulmonary fibrosis, 6 min walk test, 1 min sit-to-stand test, digital oximetry, pulse rate, eurILDreg

## Abstract

**Background**: Idiopathic pulmonary fibrosis (IPF) and progressive pulmonary fibrosis (PPF) are defined and monitored without definite exercise-based criterion, despite exertional desaturation being among the earliest functional signs of disease and an established independent predictor of mortality in IPF. The 6 min walk test (6MWT) provides robust prognostic information in IPF, yet whether it can kinetically distinguish IPF from PPF under sustained submaximal loading has not been systematically examined. We hypothesised that second-by-second oximetry during the 6MWT and the 1 min (min) sit-to-stand test (1STST) would expose phenotype-specific kinetic signatures invisible to static endpoints, and that test modality would matter. **Methods**: Fifty-one patients with IPF, 12 with PPF, and 100 with non-IPF/non-PPF ILD (reference cohort) from the European ILD Registry (eurILDreg) participated in this pilot study and completed both tests with continuous 1 Hz SpO_2_ and pulse-rate recording. Phenotype-specific desaturation and recovery slopes were derived from random-effects panel regression models. Multivariable linear regression tested whether IPF and PPF phenotypes carried kinetic and static-endpoint signatures independent of DLCO, age, sex, and BMI. **Results**: Despite substantially lower DLCO in fibrosing phenotypes (IPF 45 ± 17%, PPF 38 ± 11%, reference 55 ± 19%; *p* < 0.001), conventional exercise performance metrics did not differ significantly between groups (6MWD *p* = 0.099; 1STST repetitions *p* = 0.351). The 6MWT produced statistically indistinguishable per-second desaturation slopes in IPF and PPF (both ≈ −0.016%/s versus −0.011%/s in the reference), whereas the 1STST exposed a clear phenotype gradient (PPF −0.044%/s, IPF −0.027%/s, reference −0.016%/s; a 2.75-fold spread). PPF additionally showed a blunted chronotropic response during the 6MWT (PR slope +0.014 vs. +0.032 and +0.033 bpm/s in IPF and reference). Likelihood-ratio tests confirmed significant phenotype effects on seven of eight time-resolved trajectories (all *p* < 0.001). IPF was independently associated with greater cumulative oxygenation deficit during the 1STST (SpO_2_ AUC β = +669, *p* = 0.022), indicating excess dynamic burden beyond what diffusion capacity predicts. **Conclusions**: In this pilot analysis, continuous high-resolution oximetry identified two phenotype-related signals that were robust to the principal confounders. IPF was independently associated with a greater cumulative oxygenation impairment during the 1STST after DLCO adjustment, and PPF showed a blunted chronotropic response during sustained walking that, being pulse-rate based, was unaffected by supplemental oxygen and was not attributable to pulmonary hypertension. A phenotype gradient in per-second desaturation measurements during the 1STST was also observed but should be interpreted as hypothesis-generating, as the small PPF subgroup (*n* = 12), its greater disease severity, and supplemental oxygen use in half of its patients preclude firm attribution to phenotype. Taken as exploratory, these observations support the prospective evaluation of kinetic exercise parameters as candidate monitoring components for PPF.

## 1. Introduction

Interstitial lung diseases (ILDs) represent a heterogeneous group of disorders characterised by varying degrees of progressive impairment of gas exchange [1]. Within this spectrum, idiopathic pulmonary fibrosis (IPF) and the more recently defined progressive pulmonary fibrosis (PPF) describe clinical phenotypes associated with accelerated decline, worsening functional capacity, and increased mortality [2,3]. Despite these shared trajectories, whether the two phenotypes exhibit distinct physiological response patterns during exercise compared with other ILD entities remains poorly characterised.

Exertional oxygen desaturation is a hallmark of both IPF and PPF, frequently the first functional sign that patients experience, and an established predictor of adverse outcomes in IPF, with post-exercise pulse rate recovery carrying additional independent prognostic value [4]. Yet the question of whether IPF and PPF fail under exertion in characteristically different ways, and whether the pattern of failure depends on the loading characteristics of the test used, has not been systematically examined [5]. Recent advances in continuous digital oximetry allow time-resolved characterisation of oxygenation kinetics, including the per-second rate of desaturation during exertion and the recovery behaviour following exercise. These dynamic parameters may provide a more sensitive representation of physiological vulnerability than conventional summary endpoints such as nadir SpO_2_ or total desaturation, which compress a continuous physiological process into a small number of discrete values [6].

The 6 min walk test (6MWT) has long served as the standard field test for evaluating functional impairment and exertional hypoxaemia in ILD, whereas the 1 min sit-to-stand test (1STST) has emerged as a pragmatic alternative capable of capturing cardiopulmonary stress under short, high-intensity loading conditions [7,8,9]. The two tests appear to differ fundamentally in loading profile: the 6MWT imposes sustained submaximal demand permitting compensatory cardiovascular and ventilatory responses, whereas the 1STST apparently imposes brief intense demand that may expose reserve limitations more directly [10,11]. Whether this difference in loading profile translates into differential capacity to discriminate fibrosing phenotypes is unknown.

Against this background, the present study applied continuous second-by-second pulse oximetry during the 6MWT and 1STST in patients with IPF, PPF from the European ILD Registry (eurILDreg). By integrating panel time-series modelling with conventional functional metrics and multivariable adjustment for resting diffusion capacity, we aimed to characterise oxygenation and cardiovascular kinetics by phenotype, to identify which test modality discriminates between phenotypes, and to determine which kinetic and static parameters carry information beyond that already encoded by DLCO. Cardiovascular comorbidity is relevant to this analysis because it can independently influence both exertional desaturation and the chronotropic response. Pulmonary hypertension, coronary and other cardiovascular disease, and autonomic dysfunction, the last particularly in autoimmune/connective tissue disease-associated ILD, are recognised in fibrosing ILD and may modify pulse-rate behaviour during exercise independently of pulmonary gas exchange.

The primary endpoint was the SpO_2_ desaturation slope (%/s) during the 6MWT and 1STST, derived from second-by-second oximetry using random-effects panel time-series models, with comparative analysis of IPF and PPF against the non-IPF/non-PPF ILD reference cohort.

Secondary endpoints comprised additional kinetic parameters (SpO_2_ recovery slope, pulse-rate increase slope, pulse-rate recovery slope), conventional oxygenation metrics (SpO_2_ nadir, ΔSpO_2_ at 60 s, total ΔSpO_2_, SpO_2_ area under the curve), and functional performance measures (6MWD, 1STST repetitions). Multivariable models tested whether IPF and PPF phenotypes independently influenced oxygenation and performance outcomes after adjustment for age, sex, BMI, and DLCO % pred.

## 2. Methods

### 2.1. Study Design and Population

This cross-sectional analysis was embedded within eurILDreg, a prospective European registry established to systematically capture clinical characteristics, lung-function parameters, imaging findings, and patient-reported outcomes in ILD [12,13]. The study population comprised 163 adult ILD patients evaluated at the Center for Interstitial and Rare Lung Diseases, UGMLC, Giessen, who completed both the 6MWT and the 1STST as part of routine clinical assessment between November 2024 and March 2025.

IPF and PPF diagnoses were established according to current international recommendations through multidisciplinary case evaluation, integrating expertise from pulmonology, thoracic radiology, and pulmonary pathology [3]. Diagnostic categorisation relied on clinical presentation, HRCT imaging patterns, and, where available, histological information obtained through transbronchial cryobiopsy. Phenotype assignments were verified within the eurILDreg infrastructure to ensure diagnostic consistency and alignment with accepted classification standards. Histological confirmation, where obtained, was by transbronchial cryobiopsy only; our centre is a specialist referral centre for cryobiopsy, and surgical or forceps biopsy was not used in this cohort. All 12 PPF patients fulfilled all three 2022 ATS/ERS/JRS/ALAT progression domains within 12 months: physiological decline (FVC ≥ 5% or DLCO ≥ 10% predicted), radiological progression on high-resolution CT, and worsening respiratory symptoms, as adjudicated by prospective multidisciplinary evaluation, exceeding the guideline threshold of at least two of three criteria.

Patients entered the registry through the standard eurILDreg enrolment pathway after providing written informed consent for the scientific use of their clinical data. Eligible participants were 18 years or older, had a confirmed ILD diagnosis, and were able to complete both exercise assessments during the same outpatient visit. Exclusion criteria comprised pregnancy, acute exacerbation within the preceding six weeks, and orthopaedic or neurological conditions interfering with safe execution of the exercise protocols. Patients with cardiovascular comorbidity were not excluded; enrolment followed the standard eurILDreg pathway, and pulmonary hypertension prevalence per group is reported in Table 1.

Comprehensive clinical information (demographics, smoking history, pulmonary function results, comorbidities, imaging data, and treatment exposure) was extracted from electronic health records and standardised eurILDreg report forms. Lung-function testing followed international standards and included VC, FVC, and DLCO, all expressed as percent predicted. The eurILDreg and its predecessor, the eurIPFreg (established in 2009), are registered at ClinicalTrials.gov (NCT02951416) and DRKS (DRKS00028968). Ethical approval was obtained from the Ethics Committee of Justus-Liebig University Giessen (protocol code AZ 111/08, date of approval 4 September 2008). Of 181 patients registered at UGMLC during the study period, 18 were excluded: eight unable to complete both tests in a single visit, five with an acute exacerbation within the preceding six weeks, three with orthopaedic or neurological contraindications, and two with incomplete continuous oximetry recordings. The final analytical cohort comprised 163 patients: 51 with IPF, 12 with PPF, and 100 with non-IPF/non-PPF ILD. The approval date reflects the establishment of the registry in 2008; the eurILDreg has operated under continuous approval since, and the present dataset was collected within this approval between November 2024 and March 2025.

### 2.2. Digital Oximetry, Exercise Testing, and Data Acquisition

All 163 patients completed the 6MWT and 1STST in sequence on the same visit, with the 6MWT performed first in a 20 m indoor corridor following ATS/ERS standards [14] and the 1STST performed at least 30 min later using an armless chair, with participants completing as many full stand-sit cycles as possible in 60 s without arm support [15]. SpO_2_ and pulse rate were recorded continuously at 1 Hz using a wireless Nonin fingertip sensor throughout both tests and early recovery, generating second-by-second time-stamped data for each patient (Nonin Medical Inc., Plymouth, MN, USA). Patients on long-term oxygen therapy were assessed on their prescribed regimen; the implications for SpO_2_ comparisons are addressed in the Limitations. Arterial blood gas analysis (pH, pCO_2_, pO_2_, arterial oxygen saturation, bicarbonate, base excess) was performed at the time of exercise testing and data are available in the registry for future analyses; blood gas parameters were not included in the pre-specified kinetic analyses of the present study. Both tests were conducted under continuous supervision following ATS/ERS standards, which specify termination for severe desaturation, chest pain, intolerable dyspnoea, presyncope, or intolerable leg fatigue; a single numeric SpO_2_ stopping threshold was not applied beyond these criteria, and premature terminations were not separately recorded. Supplemental oxygen was delivered according to each patient’s established clinical prescription; flow rates and the continuous-versus-pulsed delivery mode were not captured as structured variables in the registry dataset. The interval between the two tests was a minimum of 30 min, allowing pulse rate and oxygen saturation to return towards baseline before the 1STST; the exact per-patient interval was not recorded as a structured variable.

### 2.3. Statistical Analysis

All analyses were performed in R version 4.3.1. Descriptive statistics are presented as median (IQR) and mean ± SD for continuous variables and as counts and percentages for categorical variables; group comparisons used the Kruskal–Wallis test and Fisher’s exact test as appropriate. Second-by-second SpO_2_ and pulse-rate recordings were modelled using random-effects panel regression with time (seconds) as the predictor, yielding desaturation slopes, recovery slopes, and pulse-rate slopes for each phase of both tests. To quantify phenotype-specific kinetics, phenotype (IPF, PPF, non-IPF/non-PPF reference) and its interaction with time were incorporated into the panel models. Likelihood-ratio tests compared models with and without phenotype to determine whether trajectories differed significantly between groups. This approach isolates the phenotype-specific kinetic contribution independently of test modality. Multivariable linear regression models tested which variables independently predicted oxygenation outcomes and functional performance. Outcomes included static oxygenation metrics (SpO_2_ nadir, ΔSpO_2_ at 60 s, total ΔSpO_2_, SpO_2_ AUC), slope-derived kinetic parameters (desaturation and recovery slopes), and performance measures (6MWD, 1STST repetitions). All models used forced entry with pre-specified covariates: DLCO % pred, age, sex, BMI, and ILD phenotype. Sex was retained in all models regardless of significance to ensure consistent adjustment. Borderline findings (0.01 ≤ *p* < 0.05) in this exploratory phenotype analysis should be treated as hypothesis-generating. All multivariable models used forced entry with pre-specified covariates; no stepwise selection was applied, and sex was retained in all models regardless of significance. All tests were two-sided, with *p* < 0.05 considered statistically significant. To confirm that the phenotype slope estimates were not artefacts of model specification given the small PPF subgroup, we compared each phenotype-by-time coefficient before and after covariate adjustment; the panel slopes were estimated across all 163 patients rather than within the PPF group alone. No post-hoc pairwise testing was performed; baseline group comparisons used omnibus tests only, as the analysis was focused on the second-by-second panel models rather than pairwise baseline contrasts.

## 3. Results

### 3.1. Baseline Characteristics

The reference cohort comprised non-IPF, non-PPF interstitial lung diseases, most commonly sarcoidosis (approximately 45%), autoimmune/connective tissue disease-associated ILD (approximately one fifth), and hypersensitivity pneumonitis (approximately 15%), together with smaller numbers of other entities. The 12 PPF patients had progressive disease arising from a range of underlying ILDs, predominantly autoimmune/connective tissue disease-associated ILD and hypersensitivity pneumonitis, with a minority of other fibrosing entities.

In accordance with treatment guidelines, antifibrotic therapy was offered to all patients with IPF and PPF. Antifibrotic therapy was the dominant pharmacological treatment in the fibrosing phenotypes (IPF 71%, PPF 92%; *p* < 0.001), with nintedanib used in 51% of IPF and 67% of PPF patients and pirfenidone in 20% and 25%, respectively; numbers reflect treatment active at the time of exercise testing or within the preceding three to six months. The proportion on active treatment was below 100% because a number of patients had discontinued therapy owing to adverse effects, consistent with the tolerability profile of these agents in routine practice. Long-term oxygen therapy was substantially more frequent in PPF (50%) than in IPF (14%) or other ILD (12%; *p* = 0.002). Mobility limitations were uncommon and use of walking aids did not differ significantly between groups (*p* = 0.462), as was the prevalence of orthopaedic prostheses (*p* = 0.652). Conventional exercise performance differed only numerically between groups: 6MWD was 364 ± 103 m in IPF, 310 ± 78 m in PPF, and 366 ± 100 m in the reference cohort (*p* = 0.099); 1STST performance was 21.1 ± 7.8, 17.3 ± 4.8, and 20.3 ± 7.9 repetitions, respectively (*p* = 0.351). Full baseline characteristics are provided in Table 1.

### 3.2. Time-Resolved Oxygenation and Pulse-Rate Kinetics

Panel regression models revealed an informative asymmetry between test modalities in their capacity to discriminate fibrosing phenotypes. During the 6MWT, IPF and PPF produced statistically indistinguishable per-second desaturation slopes (both approximately −0.016%/s) versus −0.011%/s in the reference cohort. During the 1STST, by contrast, a clear phenotype gradient emerged: PPF −0.044%/s, IPF −0.027%/s, and reference −0.016%/s, a 2.75-fold spread between the steepest and shallowest groups, and a 63% steeper slope in PPF than in IPF. This pattern is consistent with a physiological interpretation grounded in loading characteristics: the 6MWT is sustained and submaximal, permitting compensatory cardiovascular and ventilatory responses that may attenuate phenotype-related differences in reserve, whereas the 1STST imposes brief high-intensity loading that may unmask reserve limitations more directly.

Post-exercise SpO_2_ recovery slopes also differed by phenotype and by modality. After the 6MWT, recovery occurred at +0.014%/s in IPF, +0.062%/s in PPF, and +0.035%/s in the reference cohort; the apparently fastest recovery in PPF is most likely attributable to supplemental oxygen use during testing in half of the PPF subgroup (see Limitations). For the 1STST, the corresponding likelihood-ratio test was highly significant (χ^2^ = 128.08, *p* < 0.001), with reference recovery at +0.018%/s, PPF essentially overlapping with reference (Δ −0.0001), and IPF reoxygenation diverging from reference by Δ −0.0347%/s.

**Figure 1:**
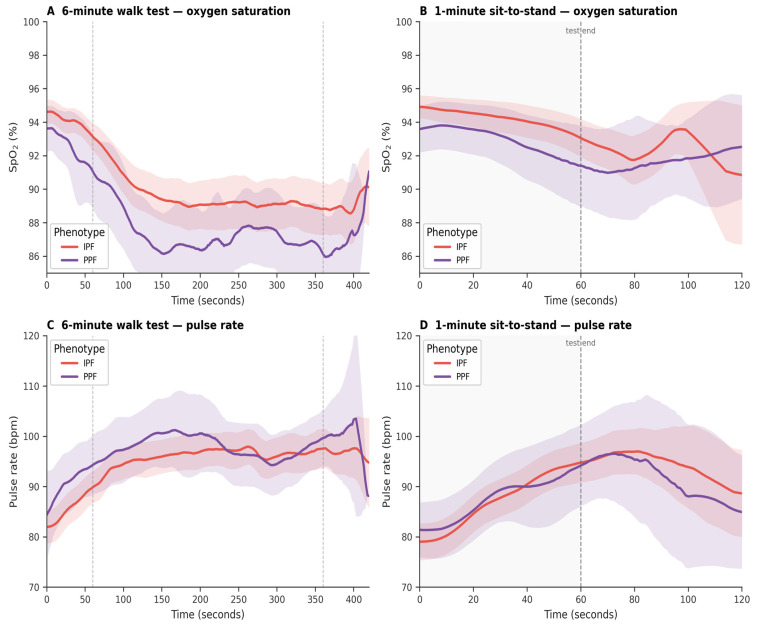
Time-resolved SpO_2_ and pulse-rate profiles during the 6MWT and 1STST in IPF and PPF, with 95% confidence bands. Shaded regions in panels (**B**,**D**) indicate the during-test window (0–60 s); the dashed vertical line marks test end. The non-IPF/non-PPF reference cohort is described in the text and is not shown here to preserve visual focus on the IPF–PPF contrast. Abbreviations: IPF, idiopathic pulmonary fibrosis; PPF, progressive pulmonary fibrosis; 6MWT, 6 min walk test; 1STST, 1 min sit-to-stand test; PR, pulse rate; SpO_2_, peripheral oxygen saturation; bpm, beats per minute; m, metres; CI, confidence interval.

Pulse-rate kinetics revealed a second phenotype-specific signal, distinct from oxygenation and concentrated in PPF. During the 6MWT, PPF demonstrated a markedly blunted chronotropic response (pulse-rate increase slope +0.014 bpm/s) compared with both IPF (+0.032 bpm/s) and the reference cohort (+0.033 bpm/s), less than half the rate of pulse-rate rise observed in the other two groups during sustained walking. This blunted response persisted, though less starkly, during the 1STST (PPF +0.230 vs. IPF +0.298 and reference +0.324 bpm/s), with all groups showing substantially higher pulse-rate loading rates during the short high-intensity test as expected. Figure 1 presents the time-resolved SpO_2_ and pulse-rate trajectories for IPF and PPF; comparison values for the non-IPF/non-PPF reference cohort are given in the text. During the 6MWT, the reference cohort showed a pulse-rate rise comparable to IPF (approximately +0.033 and +0.032 bpm/s) and steeper than PPF (+0.014 bpm/s), so the blunted PPF chronotropy is not explained by disease severity, since the milder reference group mounted a steeper, not shallower, response.

**Figure 2:**
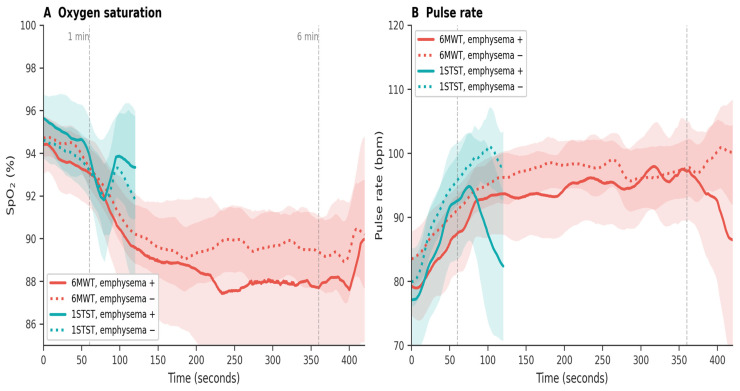
SpO_2_ and pulse-rate kinetics during the 6MWT and 1STST in IPF, stratified by emphysema status, with 95% confidence bands. Solid lines indicate the 6MWT; dotted lines the 1STST. Emphysema-positive (*n* = 17) and emphysema-negative (*n* = 34) IPF subgroups are shown. PPF was not stratified by emphysema because only one PPF patient had emphysema (*n* = 1), which precludes valid subgroup trajectory modelling. Abbreviations as in Figure 1.

Patients with emphysema tended to occupy the lower SpO_2_ and lower performance range across panels. This pattern was most apparent in IPF, where emphysema was present in 17/51 patients (33%) compared with 1/12 (8%) in PPF and 9/100 (9%) in the reference cohort. In IPF, emphysema-positive patients showed visually lower SpO_2_ trajectories and steeper decline. In PPF, the single emphysema-positive patient did not permit subgroup inference; the phenotype-level desaturation pattern in PPF should therefore be interpreted independently of emphysema status. Figure 2 stratifies SpO_2_ and pulse-rate trajectories by emphysema status within IPF; the emphysema-positive and emphysema-negative IPF subgroups showed closely overlapping trajectories, consistent with the finding that the IPF kinetic signals were numerically unchanged after adjustment for emphysema.

### 3.3. Multivariable Determinants of Oxygenation and Performance

Multivariable adjustment for DLCO, age, sex, and BMI abolished most phenotype effects on static oxygenation and performance outcomes in both tests. DLCO was the dominant determinant across all oxygenation metrics and performance measures for both the 6MWT and the 1STST; full coefficients are presented in Table 2. Phenotype (IPF or PPF) did not independently predict oxygenation nadir, desaturation depth, desaturation slope, or recovery slope after DLCO adjustment for either test. The single phenotype effect that survived full adjustment was a positive association between IPF and 1STST SpO_2_ AUC (β = +669.341, *p* = 0.022), indicating that IPF patients accumulated greater cumulative oxygenation deficit during the 1STST than their resting DLCO predicted.

Likelihood-ratio tests (Table 3) confirmed that inclusion of IPF and PPF phenotype significantly improved seven of eight time-series models for SpO_2_ and pulse-rate trajectories during the 6MWT and 1STST (all *p* < 0.001), although the additional explained variance was small (ΔR^2^ 0.0015–0.0164). The 1STST pulse-rate recovery model alone did not reach statistical significance (χ^2^ = 7.86, *p* = 0.097) and is not interpreted as a phenotype-discriminating signal. For the two findings we foreground, the adjusted between-group slope differences (95% confidence intervals from the panel models) were: for the PPF chronotropic response during the 6MWT, −0.0195 bpm/s per second (95% CI −0.0215 to −0.0175) versus the reference; and for the PPF per-second desaturation during the 1STST, −0.0286%/s (95% CI −0.0343 to −0.0229). The corresponding IPF 1STST desaturation slope was −0.0110%/s (95% CI −0.0143 to −0.0077).

## 4. Discussion

This study demonstrates that exercise physiology in IPF and PPF is more informatively characterised as a set of dynamic gas-exchange and cardiovascular trajectories than as a single capacity reduction. Distance walked, repetitions completed, nadir SpO_2_, and total desaturation each compress a continuous physiological process into a discrete summary value, and the present data show that this compression can obscure phenotype-relevant kinetic structure. When the trajectories themselves are modelled at second-by-second resolution, IPF and PPF appear to fail under exertion in non-identical ways, and the visibility of that difference depends on which test is performed.

One observation concerns the discrimination between IPF and PPF by test modality. On the 6MWT, both fibrosing phenotypes showed steeper desaturation slopes than the reference cohort, yet their slopes were indistinguishable from one another. On the 1STST, a gradient emerged across groups, with PPF showing the steepest per-second SpO_2_ decline, followed by IPF, with the reference cohort showing the shallowest desaturation. This gradient must, however, be interpreted with considerable caution. The PPF group was small, more severely impaired than the other groups, and half of its patients were tested while receiving supplemental oxygen; each of these factors independently confounds the SpO_2_ trajectory comparison, and most phenotype effects on static oxygenation endpoints did not survive adjustment for DLCO. We therefore regard the 1STST desaturation gradient as a hypothesis-generating observation rather than as established evidence of phenotype discrimination. The two findings we consider more robust are addressed below: they are, respectively, independent of resting diffusion capacity and independent of supplemental oxygen use. This pattern is consistent with how IPF and PPF fail under different demands. During sustained submaximal walking, both diseases can partially compensate through cardiovascular and ventilatory adaptation, which attenuates the reserve difference between phenotypes. Under brief intense loading, that compensation is outpaced, the reserve deficit becomes quantifiable and phenotype-specific: PPF phenotype apparently exhausts the fastest, IPF’s impairment is intermediate, and the reference cohort is the slowest. Whether this modality-dependent discrimination reflects a clinically meaningful physiological distinction or a test-specific phenomenon cannot be resolved from cross-sectional data; longitudinal studies linking these kinetic parameters to clinical outcomes would be required. Longitudinal registry observations indicating that PPF carries a higher rate of incident exertional hypoxaemia than other fibrotic ILD subtypes are at least consistent with the present pattern, though not directly comparable [14,15]. The prognostic significance of desaturation depth and post-exercise recovery during the 6MWT in IPF has been established in independent cohorts, providing broader support for the physiological relevance of exercise-derived oxygenation parameters in this population, though those findings cannot be extrapolated directly to the kinetic slope parameters reported here [15,16].

A second finding of interest is a blunted chronotropic response in PPF during the 6MWT, with a PR increase slope less than half that observed in IPF or the reference cohort during sustained walking. The reference cohort, which had higher DLCO than PPF, nevertheless showed a markedly steeper PR response, suggesting that this attenuation is not straightforwardly explained by diffusion impairment. Adjusted panel models incorporating age, BMI, FVC, emphysema status, and walking-aid use alongside the time-by-group interaction confirmed that the PPF chronotropic blunting persisted without attenuation after adjustment (adjusted β vs. reference: −0.0195 bpm/s per second, SE 0.0010, *p* < 0.001), indicating that the available confounders do not account for the observed difference. PPF encompasses a range of underlying aetiologies, including connective-tissue disease and systemic autoimmune conditions, and the PPF subgroup here showed both a high antifibrotic exposure (92%) and a substantially elevated prevalence of long-term oxygen therapy (50%). Autonomic dysfunction associated with systemic inflammatory processes, long-term oxygen therapy, and subclinical pulmonary vascular involvement could each plausibly contribute to attenuated PR responses during exercise. The present dataset does not permit mechanistic attribution; importantly, data on pulse-rate-modulating medications including beta-blockers were not systematically collected in the registry for this study period and pharmacological chronotropic suppression cannot be excluded. The small PPF subgroup limits confidence in the estimate. The adjusted panel model result provides corroboration beyond the unadjusted comparison, though the mechanistic interpretation requires dedicated prospective investigation. Current guidelines for PPF define progression through spirometry, imaging, and symptom assessment; the present finding identifies an observable physiological difference worth evaluating in appropriately powered prospective studies Notably, the unadjusted and fully adjusted estimates are numerically identical to four decimal places (both −0.0195 bpm/s per second), because the slope is estimated across the full 163-patient panel rather than within the 12 PPF patients; adjustment does not move the coefficient, demonstrating that the finding is not an artefact of an over-specified model applied to a small subgroup [3,17].

In IPF, one DLCO-independent association survived full multivariable adjustment: a positive relationship between IPF phenotype and 1STST SpO_2_ area under the curve (β = +669, *p* = 0.022). The interpretation is that IPF patients accumulated greater cumulative oxygenation deficit during the 1STST than their resting DLCO predicted. This is consistent with exercise-induced dynamic ventilation–perfusion mismatch in IPF, which may be amplified under high-intensity loading when regional heterogeneity in alveolar-capillary gas exchange is accentuated and resting diffusion measurements do not capture the full functional burden. The 6MWT did not reveal an analogous independent IPF signal, again consistent with the proposal that brief intense loading discloses physiological burden that sustained submaximal walking does not. Replication in independent cohorts will be required before this observation can be regarded as established. Recent data demonstrating that 1STST repetition count predicts mortality in fibrotic ILD and that 1STST nadir SpO_2_ provides discrimination of clinically significant exertional desaturation compared with the 6MWT provide supporting evidence that the 1STST captures physiologically relevant information in this population, though neither study examined the kinetic trajectory parameters reported here [10,18,19,20,21].

The multivariable models reveal that the IPF and PPF diagnostic labels do not add independent information to static oxygenation outcomes once resting diffusion capacity is known: DLCO dominated virtually every metric across both tests, and phenotype effects on nadir SpO_2_, desaturation depth, and performance disappeared after adjustment. This is an interpretively important finding in its own right. It means that from a static measurement perspective, knowing a patient has IPF or PPF adds no oxygenation information beyond what their DLCO already encodes. The clinical and prognostic value of phenotype classification must therefore reside elsewhere, in the kinetic structure of exertional failure, which DLCO does not predict, and in the two DLCO-independent signals this analysis identified: the IPF-specific 1STST AUC excess and the PPF-specific chronotropic blunting during walking. Whether desaturation slope and SpO_2_ AUC from the 1STST could contribute to composite functional frameworks alongside established 6MWT-derived parameters is a question that requires prospective longitudinal evaluation. The present analysis provides cross-sectional evidence that phenotype-specific kinetic differences exist between IPF and PPF and that these differences are test-modality dependent, but the design precludes stronger inference.

### 4.1. Strengths

Continuous 1 Hz pulse oximetry conducted across two complementary exercise modalities in the same well-phenotyped fibrosing-ILD cohort is uncommon in the literature; most prior work has analysed either modality in isolation and used static summary endpoints. The panel-time-series approach allows the kinetics themselves to serve as the unit of analysis. Multidisciplinary phenotype assignment within the eurILDreg infrastructure provides diagnostic consistency.

### 4.2. Limitations

Several limitations warrant consideration. The most consequential is the 50% prevalence of long-term oxygen therapy in the PPF subgroup. SpO_2_ trajectories recorded under supplemental oxygen are physiologically non-comparable to those on room air, and the apparently faster post-6MWT SpO_2_ recovery in PPF (+0.062%/s) is most likely artefactual and is not interpreted physiologically. A room-air-only sensitivity analysis would reduce the PPF subgroup to six patients, precluding reliable panel estimation; this analysis was therefore not performed. All PPF-specific SpO_2_ slope conclusions should be treated as observational. PR trajectories are not directly affected by supplemental oxygen, providing direct reassurance for the chronotropic findings. The PPF subgroup comprised only 12 patients; all PPF-specific estimates carry wide confidence intervals and should be regarded as exploratory. The 1STST pulse-rate recovery model did not reach statistical significance (*p* = 0.097) and is not interpreted as a phenotype-discriminating signal. The multivariable model in Table 2 adjusted for DLCO, age, sex, and BMI but did not include emphysema as a covariate; adjusted panel models incorporating emphysema showed that kinetic trajectory differences were numerically unchanged before and after emphysema adjustment, providing indirect evidence against emphysema-driven confounding of the kinetic signals, though a formal emphysema-inclusive reanalysis of the Table 2 AUC model was not performed. Data on PR-modulating medications including beta-blockers were not systematically collected; pharmacological contribution to the PPF chronotropic blunting cannot be excluded. Cardiovascular comorbidities including cardiomyopathy, atrial fibrillation, and chronic kidney disease were not systematically collected and their distribution across groups is unknown. Histological confirmation was not uniformly available; diagnoses were established through multidisciplinary evaluation with cryobiopsy where clinically indicated, in accordance with current international guidelines. Arterial blood gas data were collected in the registry but not included in the pre-specified analyses. The study is a single-centre and cross-sectional pilot study, precluding longitudinal or outcomes inference. Continuous oximetry is susceptible to movement artefact, particularly during the 1STST. The number of simultaneous comparisons is substantial; *p*-values in the 0.01–0.05 range should be regarded as exploratory and the IPF AUC finding (*p* = 0.022) as preliminary pending replication. Further validation in patients with milder ILD is needed. Because patients with cardiovascular comorbidity were not excluded and heart failure and arrhythmia were not separately coded, residual cardiovascular confounding of the exertional findings cannot be excluded. The requirement that patients complete both exercise tests within a single outpatient visit may have selected against the most severely impaired individuals, who might have been unable to perform both tests; the cohort may therefore be biased towards relatively better-preserved functional status, potentially most affecting the PPF group. Oxygen flow rates and the continuous-versus-pulsed delivery mode were not captured as structured variables, and premature test terminations were not separately recorded; these unmeasured factors further limit interpretation of the PPF desaturation trajectories.

## 5. Conclusions

This pilot study shows that continuous high-resolution oximetry during exercise can surface candidate phenotype-related cardiopulmonary signals in IPF and PPF that conventional static endpoints do not capture. Two signals were robust to the principal confounders. First, IPF patients carried a greater cumulative oxygenation deficit during 1STST loading than their resting diffusion capacity predicted, a signal that survived full DLCO adjustment and is consistent with exercise-induced dynamic ventilation–perfusion mismatch unmasked by brief intense demand. Second, PPF showed a blunted chronotropic response during sustained walking; because this signal is derived from pulse rate rather than oxygen saturation, it is unaffected by supplemental oxygen, and no PPF patient carried a diagnosis of pulmonary hypertension, arguing against that particular confounder. A phenotype gradient in per-second desaturation during the 1STST was also observed, but the small and more severely affected PPF subgroup, half of whom were tested on supplemental oxygen, means this gradient must be regarded as hypothesis-generating rather than as established phenotype discrimination. The 1STST is best positioned as a phenotype-sensitive complement to, not a substitute for, the 6MWT. Given that PPF is currently defined and monitored without any exercise-based criterion, these exploratory findings support the prospective evaluation of trajectory-derived kinetic exercise parameters as candidate monitoring components alongside spirometry, imaging, and symptom assessment.

## Figures and Tables

**Table 1:** Baseline characteristics of the study cohort.

Variable	IPF (*n* = 51)	PPF (*n* = 12)	Non-IPF/Non-PPF (*n* = 100)	*p* Value
Age, years	68 (64–74); 69.6 ± 7.7	70 (67–79); 72.1 ± 8.8	62 (55–73); 62.1 ± 14.1	<0.001
Male sex, *n* (%)	37 (73%)	9 (75%)	46 (46%)	0.003
BMI, kg/m^2^	25.8 (23.5–28.4); 26.9 ± 6.6	26.2 (23.7–32.5); 27.9 ± 6.3	26.7 (24.1–30.3); 27.6 ± 5.2	0.451
Pulmonary hypertension, *n* (%)	9 (18%)	0 (0%)	13 (13%)	0.272 ^†^
Emphysema, *n* (%)	17 (33%)	1 (8%)	9 (9%)	<0.001
Duration since diagnosis, months	31 (20–52); 48 ± 49	45 (32–85); 59 ± 40	68 (20–126); 87 ± 91	0.051
VC, % predicted	79 (65–90); 77 ± 18	66 (57–72); 68 ± 16	84 (69–101); 85 ± 22	0.002
FVC, % predicted	74 (63–84); 73 ± 17	64 (56–67); 65 ± 16	78 (61–93); 78 ± 21	0.021
DLCO, % predicted	45 (31–53); 45 ± 17	37 (35–38); 38 ± 11	55 (41–69); 55 ± 19	<0.001
Walking-aid use, *n* (%)	5 (10%)	1 (8%)	10 (10%)	0.462
Orthopaedic prosthesis, *n* (%)	12 (24%)	3 (25%)	14 (14%)	0.652
Long-term oxygen therapy, *n* (%)	7 (14%)	6 (50%)	12 (12%)	0.002
Antifibrotic therapy, *n* (%)	36 (71%)	11 (92%)	11 (11%)	<0.001
Current smoker, *n* (%)	5 (10%)	0 (0%)	10 (11%)	0.016
Pack-years	15 (0–30); 18.2 ± 19.9	20 (0–22.5); 19.5 ± 23.9	0 (0–15); 9.8 ± 15.6	0.008
6MWD, metres	363 (302–433); 364 ± 103	344 (216–363); 310 ± 78	383 (308–440); 366 ± 100	0.099
1STST repetitions	19 (16–25.5); 21.1 ± 7.8	17.5 (13.8–21.2); 17.3 ± 4.8	20 (16–25); 20.3 ± 7.9	0.351

*Abbreviations: ILD, interstitial lung disease; IPF, idiopathic pulmonary fibrosis; PPF, progressive pulmonary fibrosis; BMI, body mass index; VC, vital capacity; FVC, forced vital capacity; DLCO, diffusing capacity of the lung for carbon monoxide; 6MWD, 6 min walk distance; 6MWT, 6 min walk test; 1STST, 1 min sit-to-stand test. Continuous variables are presented as median (IQR) and mean ± SD; categorical variables as n (%). Group comparisons used the Kruskal–Wallis test for continuous variables and Pearson’s χ^2^ test for categorical variables; ^†^ Fisher’s exact test applied owing to low expected cell counts.*

**Table 2:** Multivariable regression models for oxygenation kinetics and functional performance.

Domain	Outcome	β IPF	*p* IPF	β PPF	*p* PPF	β DLCO	β Age	β BMI
6MWT oxygenation	Nadir SpO_2_	+0.010	0.992	−1.325	0.413	+0.193 ***	+0.030	−0.035
	ΔSpO_2_ 60 s	+0.607	0.311	−0.324	0.749	+0.034 *	+0.000	+0.018
	Total ΔSpO_2_ (360 s)	−1.035	0.222	−2.069	0.150	+0.145 ***	+0.067 *	−0.001
	SpO_2_ AUC	−704.679	0.466	+634.047	0.685	+109.830 ***	+44.954	−27.980
	Desaturation slope	−0.008	0.992	−1.138	0.356	+0.157 ***	+0.017	−0.083
	Recovery slope	−0.866	0.331	−2.005	0.184	+0.193 ***	+0.013	−0.069
6MWT performance	6MWD	+28.241	0.062	−2.882	0.911	+2.221 ***	−2.832 ***	−2.500 *
1STST oxygenation	Nadir SpO_2_	−0.833	0.251	−1.335	0.282	+0.107 ***	−0.019	−0.087
	ΔSpO_2_ 60 s	−0.262	0.646	−0.335	0.730	+0.038 **	−0.003	+0.039
	Total ΔSpO_2_	−0.770	0.127	−0.222	0.796	+0.041 **	+0.008	−0.062
	Desaturation slope	−0.226	0.649	−1.008	0.237	+0.071 ***	−0.025	−0.082 *
	Recovery slope	−0.606	0.354	−1.810	0.106	+0.102 ***	−0.023	−0.106 *
	SpO_2_ AUC	+669.341 *	0.022	+690.720	0.158	+13.454	−12.573	−22.831
1STST performance	Repetitions	+2.336	0.073	−0.135	0.952	+0.123 ***	−0.165 ***	−0.173

Regression coefficients (β) and p-values are shown for group effects (IPF and PPF, each compared with the non-IPF/non-PPF reference category) and for covariates (DLCO % pred, age, sex, BMI). Dependent variables include oxygenation parameters (SpO_2_ nadir, ΔSpO_2_ at defined time points, SpO_2_ AUC, desaturation and recovery slopes derived from random-effects panel models) and performance parameters (6MWD, 1STST repetitions) for both exercise tests. Confidence intervals for the time-resolved slope parameters, which carry the primary kinetic findings, are reported for the panel models. Negative β values for desaturation slopes indicate stronger oxygen desaturation; positive β values for recovery slopes indicate faster reoxygenation. Sex was retained in the model but did not reach significance for any outcome. Asterisks denote: * p < 0.05, ** p < 0.01, *** p < 0.001. Abbreviations: IPF, idiopathic pulmonary fibrosis; PPF, progressive pulmonary fibrosis; SpO_2_, peripheral oxygen saturation; ΔSpO_2_, change in SpO_2_; AUC, area under the curve; DLCO, diffusing capacity of the lung for carbon monoxide; BMI, body mass index; 6MWT, 6 min walk test; 6MWD, 6 min walk distance; 1STST, 1 min sit-to-stand test; RE, random effects.

**Table 3:** Global panel-model tests for the effect of IPF/PPF grouping on time-resolved trajectories.

Model	χ^2^	df	*p* Value	ΔR^2^	f^2^	Δ Slope IPF	Δ Slope PPF
6MWT SpO_2_ during	627.03	4	<0.001	0.0087	0.0113	−0.0049	−0.0058
6MWT SpO_2_ post	66.38	4	<0.001	0.0135	0.0180	−0.0210	+0.0270
6MWT PR during	401.56	4	<0.001	0.0056	0.0071	−0.0016	−0.0195
6MWT PR post	20.06	4	<0.001	0.0019	0.0021	+0.0188	+0.0458
1STST SpO_2_ during	123.08	4	<0.001	0.0121	0.0134	−0.0110	−0.0286
1STST SpO_2_ post	128.08	4	<0.001	0.0164	0.0421	−0.0347	−0.0001
1STST PR during	78.63	4	<0.001	0.0038	0.0083	−0.0258	−0.0940
1STST PR post	7.86	4	0.097	0.0015	0.0016	−0.0213	−0.0124

*Likelihood-ratio tests assessing the effect of IPF/PPF phenotype on time-dependent physiological trajectories. Random-effects panel models including the IPF/PPF group and a group-by-time interaction were compared with null models without the group effect. Reported values include the chi-square statistic (χ^2^), degrees of freedom (df), p-values, the increase in within-model explained variance (ΔR^2^), Cohen’s effect size (f^2^), and slope differences relative to the non-IPF/non-PPF reference group. Negative slope differences for SpO_2_ during exercise indicate stronger desaturation; positive slope differences for SpO_2_ post-exercise indicate faster reoxygenation. Slopes are expressed per second. Abbreviations: χ^2^, chi-square statistic; df, degrees of freedom; ΔR^2^, change in R-squared; f^2^, Cohen’s f-squared effect size; SpO_2_, peripheral oxygen saturation; PR, pulse rate; 6MW, 6 min walk; 1STST, 1 min sit-to-stand.*

## Data Availability

Data supporting the reported results are openly available via the Portal für Medizinische Datenmodelle (https://medical-data-models.org/46015, https://doi.org/10.21961/mdm:46015, assessed on 1 January 2026).
